# A Multidisciplinary Perspective of Ultra-Processed Foods and Associated Food Processing Technologies: A View of the Sustainable Road Ahead

**DOI:** 10.3390/nu13113948

**Published:** 2021-11-05

**Authors:** Francesco Capozzi, Faidon Magkos, Fabio Fava, Gregorio Paolo Milani, Carlo Agostoni, Arne Astrup, Israel Sam Saguy

**Affiliations:** 1 Department of Agricultural and Food Sciences DISTAL, Alma Mater Studiorum University of Bologna, 47521 Cesena, Italy; francesco.capozzi@unibo.it; 2 Interdepartmental Centre for Industrial Agrofood Research—CIRI Agrofood, Alma Mater Studiorum University of Bologna, 47521 Cesena, Italy; 3 Department of Nutrition, Exercise and Sport (NEXS), University of Copenhagen, 2200 Copenhagen, Denmark; fma@nexs.ku.dk; 4 Department of Civil, Chemical, Environmental, and Materials Engineering, Alma Mater Studiorum University of Bologna, 40126 Bologna, Italy; fabio.fava@unibo.it; 5 Pediatric Unit, Fondazione IRCCS Ca’ Granda Ospedale Maggiore Policlinico, Via della Commenda 9, 20122 Milan, Italy; milani.gregoriop@gmail.com; 6 Department of Clinical Science and Community Health Università degli Studi di Milano, 20122 Milan, Italy; 7 Novo Nordisk Foundation, 2900 Hellerup, Denmark; ast@nexs.ku.dk; 8 Robert H. Smith Faculty of Agriculture, Food & Environment, The Hebrew University of Jerusalem, Jerusalem 91905, Israel; sam.saguy@mail.huji.ac.il

**Keywords:** food processing and technology, precision nutrition, circular economy, sustainability, enginomics

## Abstract

Ultra-processed foods (UPFs) are negatively perceived by part of the scientific community, the public, and policymakers alike, to the extent they are sometimes referred to as not “real food”. Many observational surveys have linked consumption of UPFs to adverse health outcomes. This narrative synthesis and scientific reappraisal of available evidence aims to: (i) critically evaluate UPF-related scientific literature on diet and disease and identify possible research gaps or biases in the interpretation of data; (ii) emphasize the innovative potential of various processing technologies that can lead to modifications of the food matrix with beneficial health effects; (iii) highlight the possible links between processing, sustainability and circular economy through the valorisation of by-products; and (iv) delineate the conceptual parameters of new paradigms in food evaluation and classification systems. Although greater consumption of UPFs has been associated with obesity, unfavorable cardiometabolic risk factor profiles, and increased risk for non-communicable diseases, whether specific food processing techniques leading to ultra-processed formulations are responsible for the observed links between UPFs and various health outcomes remains elusive and far from being understood. Evolving technologies can be used in the context of sustainable valorisation of food processing by-products to create novel, low-cost UPFs with improved nutritional value and health potential. New paradigms of food evaluation and assessment should be funded and developed on several novel pillars—enginomics, signalling, and precision nutrition—taking advantage of available digital technologies and artificial intelligence. Research is needed to generate required scientific knowledge to either expand the current or create new food evaluation and classification systems, incorporating processing aspects that may have a significant impact on health and wellness, together with factors related to the personalization of foods and diets, while not neglecting recycling and sustainability aspects. The complexity and the predicted immense size of these tasks calls for open innovation mentality and a new mindset promoting multidisciplinary collaborations and partnerships between academia and industry.

## 1. Introduction

Food processing and technology have had a major impact on the availability and composition of foods throughout the human history. The earliest evidence of food processing can be traced back to prehistoric times, about 2 million years ago, when fire cooking was discovered [1]. It was not until much later, however, during the 20th century, when science and technology were applied in agriculture and food and beverage manufacturing on a wide scale; this enabled the world population to increase at an unprecedented rate [2]. From the increasing industrialization of food production systems to the introduction of the concept of ultra-processed foods (UPFs) in the 1980s [3,4], advances in food science and technology have led to a near-complete transformation of the human diet, from a traditional diet consisting mainly of home-cooked meals with minimally processed foods and food ingredients, to a modern diet with a substantial contribution of meals prepared outside home with processed foods. The majority of foods comprising modern diets are processed and have been so for centuries. Historically, the food processing and nutrition sciences have remained separate scientific disciplines, but the growing interest among policymakers, researchers, health professionals, consumers and other stakeholders in the effects of food processing on health and wellness has created a need for transdisciplinary views and practical classifications [5]. New descriptors and definitions have thus been introduced to categorize foods and beverages into different processing categories [6,7].

Food processing has historically been focusing almost exclusively on increasing palatability and shelf-life and ensuring the safety of food products. However, there is a growing need today to consider simultaneously both their health potential and their environmental footprint [3]. The future challenges of producing palatable, safe, healthy, and sustainable foods lie ahead, and need to be addressed from a multidisciplinary, integrative, and sustainable perspective. In this narrative synthesis and scientific reappraisal of available data, we provide an outlook on UPFs and discuss their effects on health and disease, also considering the paramount requirements put forth by modern societies and economies for greater sustainability. We consider advances in food science, processing technology, and innovation in the valorization of by-products and food reformulation; and discuss possibilities of redesigning existing highly processed foods with the objectives of making them healthier and sustainable. Lastly, we outline several novel aspects that can improve current paradigms of processed food evaluation and assessment.

## 2. What Is Food Processing and Which Foods Are UPFs?

Although there is no widely accepted definition, food processing can be understood broadly as any deliberate alteration in a food occurring between the point of origin (i.e., the production of raw foods or food ingredients) and the point of destination (i.e., the consumption of a final food product) [2,8]. Accordingly, modern food processing can vary in purpose (e.g., preservation, safety, quality, availability, convenience, innovation, taste, health and wellness, sustainability), and extent (e.g., as simple as: rinsing, cutting, and packaging a raw food; or as complex as: incorporating one or more food processing operations such as washing, grinding, mixing, cooling, storing, heating, freezing, filtering, fermenting, extracting, extruding, centrifuging, frying, drying, hydrogenating, concentrating, pressurizing, irradiating, microwaving, and packaging) [2]. Consumers are not familiar with most of these technologies and processes, and naturally become confused and concerned about the type and extent of “processing” of their food [5,9]. There is no doubt that processing affects the content and bioavailability of nutrients present in food [5,8], and consequently has a significant impact on consumer perception of the healthfulness of a food product. Even simple mechanical processing of a raw food decreases its perceived health effects and increases its perceived calorie content [10]. However, while some consumers opt for “minimally processed” foods because they perceive them as healthier [11], still others associate negative connotations to them because they feel these foods may pose microbiological or other safety risks [12]. This is one example of miscommunication between the scientific community, food industry, and the public, which demonstrates that several terms used to describe food processing may have a particular meaning for food engineers or food technologists, a different meaning for nutritionists or dietitians, and yet another meaning for consumers. Consumer perceptions are formulated and affected by numerous social, educational, cultural, and marketing factors, oftentimes promoted by active social media users, opinion leaders, and other influencers.

Rising concerns about the effects of industrial food processing on diet quality, health and risk of chronic diseases have led to the development of food classification systems based on food processing [6]. At least eight such classification systems have been developed, which categorise foods into different groups depending on the type, extent, place, or purpose of processing [7]. Unfortunately, these classification systems use a variety of criteria that are not always aligned with existing scientific evidence; this often results in the usage of terms surrounding processed foods that are not consistently defined and can mean different things to different people, introducing ambiguity and thereby limiting how these terms can then be used effectively in policy or advice [7]. For example, the same food processing techniques (e.g., pasteurisation or fermentation) will deem a food as minimally processed in some systems but highly processed in others [7]. In general, classification agreement between different systems is only moderate [13].

The term UPFs was introduced by NOVA [14,15], which is among the most widely used processing-based classification systems; it has been extensively applied in studies of diet quality and health outcomes [6,15], and has subsequently influenced dietary guidelines in several countries [7]. NOVA currently classifies foods and beverages into four groups: (i) unprocessed or minimally processed foods, (ii) processed culinary ingredients, (iii) processed foods, and (iv) UPFs [14,15]. UPFs are operationally defined as industrial formulations with five or more ingredients; food substances not commonly used in culinary preparations (hydrolysed protein, modified starches and hydrogenated or interesterified oils); additives used to imitate sensorial qualities of unprocessed or minimally processed foods and their culinary preparations, or to disguise undesirable qualities of the final product (colorants, flavourings, non-sugar sweeteners, emulsifiers, humectants, sequestrants, and firming, bulking, de-foaming, anti-caking and glazing agents) [14,15]. The definition of UPFs by NOVA has changed drastically over the 10+ years since its introduction, initially from referring only to the types of ingredients, to subsequently including their origin, their number, and more recently, the purpose and nature of food processing [16]. UPF-associated technologies are said to characteristically formulate cheap food imitations, which often contain little or no “whole food” [14] and have a disrupted food matrix leading to the loss of the natural “matrix effect” [17]. However, both of these terms are not precisely defined [7], making identification of UPFs not a straightforward exercise. The complexity of industrially produced foods is such that without precise category definitions and mutually exclusive categories, the potential for misclassification is high [13], so that agreement between independent reviewers in the ranking of a food as UPF or not is only moderate (particularly when it comes to mixed dishes) [18]. These observations indicate that there is room for improvement, for instance, by developing standardized food audit tools to identify UPFs with improved test validity (inter-rater reliability and test-retest reproducibility) [19]; but also highlight the need for ongoing development of the food processing evaluation and classification systems themselves.

Some common UPFs are: carbonated soft drinks; sweet, fatty or salty packaged snacks; candies (confectionery); mass produced packaged breads and buns, cookies (biscuits), pastries, cakes and cake mixes; margarine and other spreads; sweetened breakfast cereals and fruit yoghurt and energy drinks; pre-prepared meat, cheese, pasta and pizza dishes; poultry and fish nuggets and sticks; sausages, burgers, hot dogs and other reconstituted meat products; powdered and packaged instant soups, noodles and desserts; and baby formula [14]. UPFs include not only “junk foods” but also foods marketed as healthy, such as light, vegan, organic, or gluten-free products [17]. For example, most plant-based dairy and meat substitutes are UPFs [20]; as a result, UPFs contribute about 40% of total energy intake in vegetarian and vegan diets [21]. It is also worth noting that most recent attempts to develop innovative food products at the intersection between food science, processing technology, and sustainability, such as plant-based meat (e.g., https://www.beyondmeat.com/), cultured meat (e.g., https://www.aleph-farms.com/), plant-based milk (e.g., https://www.ripplefoods.com/), cultured milk (e.g., https://www.biomilk.com/) and zero eggs (https://www.zeroeggfood.com/), are likely to be classified as UPFs. 

## 3. Relationship between Food Processing, Nutritional Value, and Health Effects

The food manufacturing industry—as a whole—is driven primarily by the motive to increase profits, which requires minimizing costs across all stages of production; this is true regardless of whether it produces highly processed potato chips (a UPF) or extra virgin olive oil (a minimally processed food). In some cases, this may be achieved by the use of cheaper alternative food ingredients whenever possible and to the extent possible and, on some occasions, this practice can result in UPFs with unfavorable health effects. An example is the partial hydrogenation of vegetable oils (which are liquid at room temperature) to convert unsaturated fatty acids to saturated fatty acids and make these oils more solid or “spreadable”. This process was used in the food industry from around 1920 to produce solid margarines that were cheaper and were marketed as healthier than butter, and widely used for baking, cooking and frying [22,23]. In 1993, the first warning signs suggested that the resulting high content of trans fatty acids had severe adverse effects of cardiovascular health, and the high intakes of trans fat from popular foods were found to be partly responsible for the increasing cardiovascular disease mortality; subsequently, trans fat was restricted or banned in several countries [22,23]. Still, it is important to realize that the type and extent of food processing do not necessary correlate with the health potential of the final product. There are, in fact, many emerging processing technologies that can improve the nutritional value of food products, e.g., by mitigating the loss of nutrients [8,9].

UPFs are negatively perceived by part of the scientific community, the public, and policymakers alike, to the extent they are sometimes referred to as not “real food” [24]. Much of this bad press has been based on the concurrent increases in the consumption of UPFs, the prevalence rates of obesity, and the risk for chronic non-communicable diseases (NCDs) attributed to diet [25,26,27,28]. Although there is considerable overlap between the processing classification and the healthfulness ranking of foods [29], processing and nutritional value are not linearly related to each other. Instead, these two dimensions of food evaluation metrics intersect, as approximately one-fourth of foods with high nutritional value, which rank among the healthiest, are highly processed or UPFs [29,30]. Importantly, on a global scale, among 15 dietary risk factors examined, a high sodium intake and low intakes of whole grains, fruits, vegetables, and nuts/seeds rank at the top for both number of deaths and disability-adjusted life-years; whereas high intakes of sugar-sweetened beverages and processed meats rank at the very bottom [25]. This suggests that, when it comes to assessing the healthfulness of foods and the impact of diet on human health and disease, traditional nutrient-based systems that focus on nutritional content, quality and safety characteristics may perform better than processing-based evaluation systems that focus on industrial formulation [31]. It is, of course, likely that considering both the nutritional content and aspects of food processing in combined food evaluation metrics, within a more holistic approach to food and diet, will be more informative [32]; attempts in this direction are currently under way (see Section 4.2).

The majority of UPFs available for consumption nowadays have long shelf-life and can be consumed in any place and any time (“ready-to-eat” or “ready-to-heat”) [33]. Breakfast cereals, crackers, and cheese spreads, but also yogurt and granola bars, are all ready-to-eat food products with largely similar extent of processing [9], although several of these may or may not be classified in the same category by different classification systems, or even by the same classification system depending on their formulation. Purely from the perspective of food processing technology, however, many of the foods utilized in ultra-processed diet paradigms (e.g., white breads, potato chips, apple sauce, refined grain products) are no more extensively processed than olive oil that requires processing of olives to disrupt the natural food structure and remove all fibers, or dark chocolate that typically contains a half-dozen or more refined ingredients, which are generally considered healthy foods [34]. UPF-associated technologies are perceived as adding and combining substances coming from food cracking/fractionation and other techniques that are not commonly used in home cooking. By extrapolation, all homemade cakes, pizzas, French fries, biscuits and jams are not UPFs, regardless of how they are prepared [7,30]. This is because cooking at home is considered a minimal or moderate processing approach to food preparation, and thus inherently healthier [14,15]. However, some studies have demonstrated that home-cooked foods and home recipes are not consistently higher in nutritional quality and may even be worse than ultra-processed alternatives [35].

At present, UPFs dominate the current food supply and provide more than 50% of dietary calories in most high- and middle-income countries, and increasingly also in low-income countries [24,28,33,36,37,38,39]. Available UPFs typically combine many adverse dietary factors including high energy density, highly rewarding palatability, large portion size, low micronutrient and fiber contents, high free sugar and salt contents, poor quality fat and protein, and high glycemic index carbohydrate and glycemic load [33,40,41,42,43]. Most, if not all of these factors have long been known to be independently associated with adverse health outcomes, including obesity and several metabolic abnormalities such as insulin resistance, dyslipidemia, and hypertension [44,45]. There is indeed growing evidence from association studies linking increased consumption of UPFs with higher risk of obesity and NCDs [36,38]. Furthermore, results from the prospective French NutriNet-Santé study in more than 100,000 subjects over 4–6 years suggest that greater consumption of UPFs is directly associated with greater risk for weight gain and developing overweight and obesity [46], and greater risk for type 2 diabetes (which is largely but not fully explained by weight gain) and cardiovascular diseases [29], independent of total calorie intake, baseline body mass index, and several other established nutritional risk factors. Similar results have been obtained in other smaller prospective studies, including the UK Biobank [39,47] and the SUN (Seguimiento University of Navarra) cohort study in Spain [48]. What is not clear, however, is whether processing itself is responsible, or whether the actual unfavorable nutrient profile of these UPFs or even other factors are to blame [35,37]. As an example, pure fruit juice is a relatively fiber-poor form of carbohydrate with destructed matrix compared to whole fruits, and juicing can be done by simple processing in the kitchen at home. Pure fruit juice has lower satiating effect and promotes ad libitum overconsumption of calories compared to whole fruits [49], but consumption of pure fruit juice has also been linked with reduced risk for NCDs [50]. This suggests that neither the food matrix itself, nor the nutrient content, or the extent of processing of food can be solely responsible for the observed health effects.

There are not many carefully controlled interventional human studies providing insight into possible cause-and-effect relationships between consumption of UPFs and health outcomes. Ideally, these studies should take into account various sociodemographic and environmental variables that are beyond the scope of the present review but are increasingly considered to contextualize the associated health outcomes. In one recent cross-over feeding study, twenty subjects with overweight or obesity were confined in a metabolic ward and provided with an ultra-processed diet and an unprocessed diet for two weeks each, in random order [51]. The meals were designed to be matched for presented calories, energy density, macronutrients, sugar, sodium, and fiber, and were consumed ad libitum. When on the ultra-processed diet, subjects chose foods with higher energy density (despite that presented meal options were matched for this), ate faster, and consumed significantly more calories (~500 kcal/d), carbohydrate, fat, and sodium than when on the unprocessed diet; and gained weight [51]. Therefore, at least in the short-term, UPF-based diets facilitate overeating compared with unprocessed food-based diets. Still, one cannot exclude alternative explanations. Given these subjects were habituated on a diet comprising primarily UPFs before the intervention, it is possible they simply over-ate the foods that were more familiar to them, particularly when an abundance of these foods became available at no cost [34]. Of note, body weight drifted downwards on the unprocessed diet, which suggests that at least some of the difference between diets was because subjects under-ate the unprocessed foods; on this instance, the participants were presented with unfamiliar foods in a raw (or less processed) form that required more effort to consume, and therefore ate slower and overall less [52]. Furthermore, given that energy intake on the UPF diet decreased linearly with time throughout the study but did not change on the unprocessed diet, differences between diets gradually diminished [51], so that long-term extrapolation of these results should be made with caution. This ground-breaking trial was not designed to establish the mechanism by which the UPF-based diet caused excess ad libitum energy intake, and all these possibilities need to be experimentally tested in future studies.

The faster eating rate alone [51] could be responsible for the greater energy intake on the UPF diet, independently of any effects from processing, differences in food type, form, or matrix [52,53]. Most of the studies included in a previous meta-analysis that reached this conclusion examined the effects of eating the same food and in the same form and matrix at slow or fast rates [53]. The effect was actually more pronounced in studies in which the eating rate manipulation was achieved by verbal instructions, computer feedback cues, or controlled food delivery; rather than in studies in which the eating rate manipulation was achieved by altering the matrix and form of the food [53]. Furthermore, in a pooled analysis of data from five published studies that measured energy intake rates across a total sample of 327 foods, consuming foods at higher rate was related to higher total energy intake independent of the extent of processing [54]. Average energy intake rate increased from unprocessed (~36 kcal/min), to processed (~54 kcal/min), to UPFs (~69 kcal/min), but within each food processing category there was a huge variability and considerable overlap (from 2 to 240 kcal/min for unprocessed foods, from 6 to 188 for processed foods, and from 0 to 249 for UPFs) [54]. Accordingly, future research should investigate the mechanisms by which UPFs promote higher energy intake and the possible independent effects of processing. It is certainly possible that some processing techniques lead to food matrices that demand less chewing and therefore, facilitate eating at a faster rate and overall, more.

Besides changes in energy intake, another potentially important route by which consumption of UPFs may influence body weight homeostasis is energy expenditure. Diet-induced thermogenesis is the increase in postprandial energy expenditure above baseline that occurs upon eating, because of absorption, digestion, transport and storage of ingested nutrients but also heat production; thus, it may well be affected by the composition and formulation of the food. A lower diet-induced thermogenesis would result in lower total energy expenditure and would promote the induction of positive energy balance, which could lead to weight gain and obesity. Indeed, some studies found that UPF-based meals induce a significantly lower postprandial energy expenditure compared with isocaloric and iso-macronutrient meals (i.e., same carbohydrate, protein and fat amounts) consisting of unprocessed or less processed foods [55]. However, others found exactly the opposite [56]. These discrepant observations indicate that greater degrees of food processing and food engineering need not necessarily result in nutritionally poor food products with adverse metabolic effects (i.e., lower postprandial thermogenesis). Whatever the case, results from such single meal test studies cannot be used to interpolate changes in total daily energy expenditure under free-living conditions.

Although greater consumption of highly processed foods has been associated with excess body weight, hypertension, dyslipidemia and other features of the metabolic syndrome and, consequently, increased risk of NCDs and total mortality [3,57,58,59,60], conclusive evidence that unequivocally demonstrates causal links between UPFs and occurrence of NCDs is lacking [60]. As tempting as it may be to suggest that the existence of many observational studies (both cross-sectional and longitudinal in nature) converging towards the same conclusion is sufficient to make recommendations for public health regarding food consumption, the history of medical research is full of case reports where inappropriate study designs have led to erroneous conclusions with lasting consequences for health policy and clinical practice [61]. Furthermore, one cannot deny the existence of some paradoxical and apparently contradictory findings. Vegetarians and vegans have more favorable risk factor profiles and better health outcomes than omnivores in almost all observational studies [62,63]. Yet the contribution of UPFs to total energy intakes in vegetarian and vegan diets is significantly greater than in omnivorous diets [21], as the majority (>90%) of plant-based meat and dairy alternatives are industrial formulations created from the combination of multiple ingredients not typically used in home cooking [20]. Accordingly, whether specific food processing techniques are responsible for the observed links between UPFs and various health outcomes remains elusive and far from being understood.

## 4. Food Processing and Technology: State-of-the-Art and Future Innovation

### 4.1. Current Status

The current definitions of highly processed foods and UPFs do not adequately reflect the intensity of the technological process (e.g., pasteurization, sterilization, extrusion), the processing conditions (e.g., temperature, pressure, time, oxygen), and the loss of food matrix effects (e.g., whole fruit, chopping, mincing, juicing). A PubMed literature search of the terms “highly processed food” or “ultra-processed food” appearing in the abstract yielded a total of 817 unique hits (25 July 2021), but when combined with terms such as “matrix” (15 hits) or “thermal processing” (4 hits) or “pasteurization” (0 hits) or “sterilization” (2 hits) or “extrusion” (0 hits), the search returned minimal results. This indirectly suggests that even though the various definitions of processed foods refer to various types of processing used by the industry, relevant research into how exactly these techniques affect the properties of the final food products, and how the various processing conditions and the continuous refinement of these techniques can affect the composition and healthfulness of these foods, is practically non-existent; and is therefore not considered in any of the classification systems. In fact, the technological process is rarely considered in studies of nutritional epidemiology and can be found mainly in the form of binary comparisons [64], such as red meat versus processed meats [65], fresh fruit versus fruit juices [66], fruit juices versus sweetened beverages [67], milk versus yogurt or cheese [68], and whole-grain versus refined cereals [69]. These studies tend to show that compared with the minimally or less processed alternative, the more processed version of the food is less protective against NCDs [58].

### 4.2. The Need for a Comprehensive Food Evaluation Metric

Several classification systems that categorize foods according to their “level” of processing have been used to predict diet quality and health outcomes and inform dietary guidelines and product development. In a recent analysis of these systems and the criteria used to classify foods, it was concluded that the classification criteria are ambiguous, inconsistent and often give less weight to existing scientific evidence on nutrition and food processing effects [7]. A qualitative approach for improving current processing-based classifications (and particularly, the NOVA) has been suggested [3]; this approach takes into account the nature, quantity, function, and degree of transformation of the ingredients or additives, and the loss of the food matrix effect, in order to achieve a more holistic and realistic classification [3]. Consequently, the Siga (“go forward” in Portuguese, meaning “go further” or “improve the existent”) classification was developed by combining the four NOVA groups with four new subgroups [64]. This classification considers the impact of processing on the food/ingredient matrix; the contents of added salt, sugar and fat; the nature and number of “markers of ultra-processing” (termed MUPs); and the levels of at-risk additives. The eight new food categories are: unprocessed (A0); minimally processed foods (A1); culinary ingredients (A2); nutritionally-balanced processed foods (B1); high-salt, high-sugar or high-fat processed foods (B2); nutritionally-balanced UPFs (C0.1); high-salt, high-sugar or high-fat UPFs (C0.2); and UPFs with more than one MUP (C1) [64].

The need for quantitative food processing evaluation metrics has also been described [3]. The necessity to shift from qualitative to quantitative food classification systems according to their degree of processing through the development of a quantitative technological index, including both “matrix” and “composition” effects (and not only nutritional content), has been increasingly realized over the last decade [3,70]. To this end, further studies, both epidemiological and experimental, will be required to investigate the relative contribution of nutritional composition, food additives, process- or packaging-related contaminants, and food matrix modification on various health outcomes [46].

The possibility that the food matrix can influence delivery and bioavailability of nutrients and other bioactive components, as well as gut microbiota profile; and also potentially lead to weight gain when energy-providing nutrients or other components are delivered faster because of altered food structure, has been put forth [71]. Even with identical chemistry, food structure may result in major differences in biological and health outcomes. With evidence suggesting that food structure strongly contributes to the “matrix effect”, concern has grown about the health effects of highly processed foods. To improve knowledge on food structure, food composition tables will need to provide information on particle size and viscosity, but also list the various food ingredients [71]. Additionally, genetic influences may further contribute to explain the wide interindividual variability in the metabolic responses to meals comprising foods with similar structure and matrix [72]. The transmission belt between food matrix, interindividual digestive enzyme variability, and gut microbiota may help explain the biological possibility of paradoxical metabolic derangements, such as the glucose intolerance manifesting in animals and humans with the use of non-caloric artificial sweeteners [73].

Processing influences not only the composition of the food but also its structure and matrix. The matrix furnishes support, architecture, or gives a food its form, thickness, density, hardness, porosity, color, and crystallinity [3], all of which are very important attributes for consumer acceptance. Each food has its own matrix, which determines the bioavailable fraction of every ingredient, as well as the satiety potential of the food itself, with solid foods being more satiating than semisolid and liquid foods [74]. Accordingly, more unstructured matrix is consistently being ranked lower in its satiety effect [75,76,77]. Numerous other nutritional attributes and quality aspects (e.g., vitamin bioavailability) are affected by the food matrix. Studies have demonstrated the importance of food structure during the digestion of plant foods. For instance, the amount of lipid released from almond tissue matrix and the fatty acids produced from lipolysis have been found to vary substantially depending on the structure and degree of processing of the almond kernel [78].

When referring to the various processing technologies that can lead to the production of UPFs, notably based on food fractionating and extrusion-cooking, the word “ultra” seems to refer to the extreme destructuring action. However, rather than destructuring, a function-driven structure rebuilding would be a more appropriate concept for processing technologies. Since the structure of UPFs can be even more complicated and heterogeneous than a minimally processed food, processing-based food classification systems such as the NOVA should focus more on the complex composition of the foods rather than on the possible matrix-destructive effect of the processing technologies. While, for instance, it is sufficient to indicate the presence of apples and sugar in a homemade jam, for a jam produced in the industry the fruit can be recomposed by mixing more ingredients, still originating from the same raw materials but included in the optimal formulation, which is both functional and advantageous for product palatability and stability. This typically results in a significant increase in the number of fractionated ingredients listed on the food label that, subsequently, classify the food as a UPF.

### 4.3. Delineating a New Paradigm

Two important goals deserve implementation: (i) standardise classifications based on the level of food processing to promote comparability [79], and (ii) disentangle the contribution from different aspects of food processing and identify which aspects of processing can result in adverse health effects [80]. Most, if not all of the current processing-based classification systems are limited because of several reasons, such as: (i) foods with the same composition but different matrices or structures, produced by different processes (e.g., extrusion, sterilization) and processing conditions (e.g., time, temperature, pressure), have different nutrient bioavailability; (ii) the health potential of food is due to a wide array of complex factors not fully encapsulated in current constructs; (iii) innovative technologies classified as non-thermal processes (e.g., high pressure processing, pulse electric fields, cold plasma, ultraviolet) have been developed and some are already being implemented today in the food industry offering unique nutritional and consumer benefits; (iv) the lack of integration of recycling and sustainability aspects; and (v) the lack of consideration of the unabated progress in digital technology that offers new opportunities to monitor biomarkers that can help design diets for optimal health of individuals or specific subgroups of the population (“precision nutrition”). Although this will not be an easy task, and will require input from transdisciplinary research teams, considering all or most of these factors has the potential to result in better and more informative food evaluation metrics than those currently available.

Therefore, a new paradigm of food production, assessment and evaluation is required, founded on three main pillars: (i) “enginomics” [81,82,83]; (ii) “signalling” [84]; and (iii) “precision nutrition” and related health and wellness [85].

*Enginomics.* Enginomics (engineering + omics) refers to the integration of the effects of food processing and structure design on nutrient bioavailability (host/microbiome) and omics (e.g., genomics, proteomics, lipidomics, metabolomics, transcriptomics, epigenomics, microbiomics). It recognises the multidisciplinary and innovative approach being undertaken in food technology and engineering, and places a holistic focus on health within the context of an environmentally sustainable and socially responsible model. Enginomics comprises several pillars [82,83]:

1. Human internal unit operations (e.g., digestibility, gastric aspects, targeting, bioavailability, bioaccessibility)
2. Health and wellness (e.g., medicine, brain, biology, microbiome, pro- and prebiotics, nanotechnology, biotechnology) and nutrition (e.g., personalization, special group needs, prevention, satiety)
3. Food and product engineering (e.g., properties, composition, new resources, structure, design, material science, packaging)
4. Manufacturing (e.g., processing, waste and water management, environment, compliance, regulations, developed and developing countries)
5. Consumers (e.g., safety, acceptability, special needs, sensations, pleasure, cost, convenience)
6. Social responsibility (e.g., food security, feeding the world, sustainability, growing population, aging, water and land scarcity, ethics, moral values)

The matrix is an essential and integrated part of the food structure, and the combined effects of the bioavailability of both nutritive and non-nutritive components, to both the host and the microbiome, and subsequent effects in the production of microbiota metabolites should also be considered. For instance, it was previously recommended that future research should address the impact of processing methods on the structure of dietary fiber, the variation in the composition of gut microbiota, and the metabolism of fiber [86]. Also, the intentional modification of food matrix and structure to control the bioavailability of pharmaceuticals and nutraceuticals has been recommended within the context of dietary prevention and treatment of NCDs [87]. “Biomimetics” is a new field that takes inspiration from nature and offers solutions for the future by designing and producing natural-like food matrices and structures (which may be considered highly processed “food imitations” by some current classification systems) for improved health and well-being; more often than not this requires a combination of innovative technologies [88]. With the proliferation of 3D printing and its utilization in food production [89,90], biomimetics—which has the potential to also integrate recycling and sustainability aspects into the food production process—becomes a very plausible and realistic option.

*Signalling.* Some food processing techniques used in the industry but also some others used in home cooking can lead to untoward health consequences under certain conditions. High level pathogen-associated molecular patterns (PAMPs) are toxins excreted by pathogenic bacteria (e.g., *Pseudomonads, Enterobacteriaceae*) that may develop during processes employed in the kitchen or applied commercially for minimally processed and refrigerated foods (e.g., products containing ready-chopped vegetables, minced meat, ready-to-eat meals stored at 4oC). Signalling caused by PAMPs or their derivatives formed during processing may stimulate innate Toll-like receptors (TLR2 and TLR4), leading to strong immune reactions associated with inflammation, type 2 diabetes, atherosclerosis, and increased cardiometabolic risk factors [84]. Therefore, safe processing conditions are necessary regardless of the place and extent of food processing, as even apparently minimal processing food preparation techniques (e.g., dicing, mincing) could pose health risks.

*Precision nutrition.* Tailoring the diet to specific physiological characteristics of individuals, or specific subgroups of the population with homogenous biomarker profiles, has attracted both academic (e.g., https://preventomics.eu/) [91,92,93] and industrial attention from start-ups (e.g., https://www.daytwo.com), some of which make use of artificial intelligence to personalise the diet (e.g., https://lifedata.ai/digital-health/personalized-nutrition). These technological advancements are prime candidates for shaping the future of food production, diet design, and dietary recommendations.

## 5. Opportunities for UPFs within the Frame of Circular Economy

The potential for using innovative processing technologies to create healthy and sustainable foods has not been adequately materialized. This is particularly relevant as it is often assumed that sustainable food is—in itself—optimal for health. Possible ways in which sustainability of the modern food production systems can be combined with healthfulness of the food products should be explored, regardless of the production practices of raw materials. These practices need to meet the growing demand for nutritious, safe and sustainable food; and thus need to rely on management models with a more efficient water consumption and responsible utilization of all natural resources. Additionally, from the same raw materials, sustainable processing technologies can be used to enrich foods with nutrients and other bioactive molecules with putative health effects. Sustainability, therefore, should be holistically included as an integral part of the UPF domain.

### 5.1. Current Challenges of Sustainable Nutrient Sources in the Food Chain

The food value chains are considered sustainable when production and distribution systems are organized to ensure global economic and social development, while retaining sufficient planetary resources available for future generations. In 2014, the Food and Agriculture Organization defined the three main effects that production processes have in the food value-chain system; these included economic, environmental, and social impacts [94]. Regarding environmental impact, the parameter that needs to be optimized during the transformation of raw materials into food, examined from a “farm to fork” perspective, is the natural resource footprint, such as the consumption of biological resources and water, considering those directly found in the final food products and those necessary for their production [95]. In parallel, food loss, from the primary production to the food market, together with food waste, produced between the distribution and consumption points, should be minimized, and this largely depends on the product shelf-life [96]. Production of UPFs has been reported to be associated with intensive agriculture/livestock, and consequently potentially threatens several dimensions of food system sustainability due to the combination of low-cost ingredients and increased consumption worldwide; however, the impact of UPFs on greenhouse gas emission is not greater than that of less processed alternatives [97]. Moreover, advancements in food processing technologies can affect this source of dispersion that generates a significant impact on the entire supply chain, and thereby offset the potential threat to sustainability and biodiversity [97].

Sustainability, as a concept, is rather complicated as it must consider the economic, the social, as well as the environmental costs of food production, together with the resulting benefits. Avoiding food production altogether is certainly the situation with the lowest possible environmental footprint but this does not bring any benefits to food availability and food security. Producing the same food at a lower cost by using renewable raw materials, with lower social and environmental impacts, makes the system more sustainable. The benefit/cost ratio can increase when the denominator is decreased, but it can be maximized only when the numerator is also increased concurrently. This can be done by improving the nutritional quality of the food, for example by enriching it with a novel food component. For instance, the extrusion process almost doubles (from 10% to 17%) the amount of soluble dietary fibers extracted from wheat, which contains 40% total fibers [98]. One can therefore enrich food with soluble dietary fibers to the expense of cellulose contained in the bran, which also makes the product less palatable. Extrusion has a cost, but its application yields final products of much higher quality, and therefore it balances-out the higher cost.

The need to produce more and healthier food through more sustainable production systems drives the food industry to design new products and processes, to address the demand for innovation expected by both the consumers and the market, and to involve all stakeholders at multiple levels within an economic framework. Lifestyle changes and the modern way of living are pushing for “convenient” food availability [99]. In addition, the growing scientific knowledge increases demand for healthy foods, which must be made available at affordable cost as the population at risk of poverty is growing and constitutes a considerable portion of the society [100]. Safeguarding access to nutritious diets among the poorest households (including access to fortified foods) needs to be expanded. Such motivation drives the creation of new food products that require careful cost optimization, from the adopted raw materials up to the processes selected to make the new formulations effective, affordable, and attractive [101].

The use of processing technologies is indispensable to transform low-cost sources into high-value ingredients, and deliver food products with an optimized composition that are acceptable by the consumer. Circular economy—defined by the EU as a model of production and consumption that extends product life cycle by sharing, leasing, reusing, repairing, refurbishing and recycling existing materials and products for as long as possible [102]—can valorise unexploited sources that are available at low-cost and not entered in the food value chain because they are not in the proper form. By-products play an important role in the design of innovative food products, especially those fortified with specific bioactive molecules, because they typically represent a low-cost source [103]. By-products and side-streams coming from several food production pipelines that can be safely collected and stored represent sources of prominent high-value ingredients and natural products, which can be exploited in the formulation of novel foods. This, in turn, can contribute to a systemic and inclusive economic, environmental, and social regeneration of the agrifood systems, with the production of novel foods characterized by high nutritional value, economic affordability, and environmental sustainability. This process may effectively create new market opportunities and competitiveness and reduce the environmental burden associated with the disposal of secondary streams, which are currently poorly valorised (mainly limited to fertilizer or bio-fuel production). It is worth noting that ingredients and products based on circular economy are not by definition UPFs; hence the future definition of UPFs should take this into consideration.

By considering the links between processing and fractionation of the raw matter, it appears that food chains including such technologies are also responsible for producing waste, which would not be generated if foods were not deconstructed to that extent. However, any type of food production generates waste, even at home, because not all parts of the raw materials and ingredients are edible. It is estimated that fruits and vegetables produce significant waste, composed mainly of seed, skin, rind, and pomace, which constitutes about 25 to 30% of the whole commodity [104]. In fact, most plant-based food manufacturing industries, including minimal processing ones, face the problem of waste that is currently mainly destined for compost or energy production. Even in the case of bran, industries have no interest in separating it from the flour, because eliminating it has a cost whereas retaining it increases the mass of the final product. The refining of flour is a market request that leads to the production of waste or, at the very least, products used as animal feed. Conversely, through innovative processing technologies, ingredients with nutritional value such as soluble dietary fibers, or simple valuable natural compounds, can be extracted from waste.

### 5.2. Valorisation of By-Products to Produce Healthy UPFs

Carbohydrates, lipids, proteins and other polymers present in by-products may be increasingly converted into the corresponding oligomers or monomers such as fatty acids, amino acids, simple sugars, dietary fibers, antioxidants, and other micronutrients, through tailored biological treatments [105,106]. The purer and freer of contaminants these simple components are, the higher the chance they may advance in a scale of natural value as bioavailable chemicals amenable for utilization, initially by the food industry but also by the pharmaceutical, nutraceutical and biocosmetic industries. The exploitation of food-grade ingredients in optimized food matrices is a valuable opportunity to enrich products and to extend effective concentrations.

After the selective recovery of target compounds via established membrane and chromatographic technologies, some other unrefined compounds left in the raw matrix can be further valorised via tailored enzymatic bioconversion or microbial fermentation, with the production of well-characterized natural fine chemicals that can be used as specialties in various industrial applications [105,106]. This additional sequence of cascading operations avoids contamination from chemical catalysts or genetically modified microbes and related enzymes [107], thus delivering products suitable as ingredients for human consumption and other applications. An example of exploitable by-products is represented by vegetation waters that are separated from the oil during the pressing of the olives [108], and the mesocarp (“albedo”) from the orange peel that is left over after removing the surface layer (which is rich in essential oils) and the juice (for drink production) [109]. It is worth noting that in the orange juice industry, more than 50% of raw material becomes by-products that are rich in active compounds and have high nutrient content [110]. The leftover material resulting from the sequential recovery steps can be valorised, together with the biowaste streams coming from the same or related food industries, via multiproduct biorefinery schemes, where sequential chemical and biological steps may convert the biogenic carbons into a variety of chemicals and other materials that are of prominent interest for the chemical, cosmetic, textile and energy sectors [105].

Dietary fibers are ingredients that face an emerging demand from the food market, stimulated by a growing scientific knowledge highlighting their health potential [111]. One of the main sources of fibers is wheat bran, obtained as a waste from the cereal industry because whole-wheat flours are less palatable. Processing technologies may allow the reintegration of these fibers into bakery products which can become tastier through fermentative processes. Therefore, the stabilized bran can follow a threefold recovery path. The first is the colonization with *Lactobacilli*, for producing probiotic-enriched fibers; the second is enzymatic treatment for conversion of cellulose into oligosaccharides with prebiotic potential, which can be used as ingredients in many food preparations; and the third is the recovery of ferulic acid, an abundant antioxidant compound with an unpleasant bitter-astringent taste, and its conversion, by fermentation with Pseudomonas fluorescens, into a naturally-equivalent of vanillin [109]. This generates an aroma flavour that is much appreciated by consumers in the confectionery industry, a sector where artificial flavours are no longer advocated. These processes may add sensory value to the well-documented nutritional value in the re-utilization of dietary fibers [112]. Obtaining dietary fibers from bran is not the only decisive process for the successful production of fortified bread products. Ingredients should be advantageously added into newer formulations, in doses and forms that can add detectable health benefits to the original food matrix, yielding a new matrix, while simultaneously meeting requirements of innovative yet natural food forms [113].

Technological processes must be perpetually optimized to convert ingredients into edible food, in a form that is acceptable by the consumer. An unpalatable or sensorially unattractive food will not succeed in the market, even if it has excellent nutritional properties; without a proper flavour, the product is likely to be unsuccessful. For this reason, reaching a desired sensory structure in the final product is of paramount importance for its success. Even using the same ingredients, but with different processing parameters, very different tastes can be obtained that are readily appreciated by expert sensory panels [114] and consumers. For example, inadequate formulations and processing conditions may result in types of bread with a more intense bitter taste of the crumb. For instance, the optimization of the parameters of the processing technologies was one of the objectives of the CHANCE research project, funded by the EU, aiming at obtaining bakery products for populations at risk of poverty. The products were enriched with fibers while using low-cost ingredients [100]. Without an optimization of the technological processes, the bread developed would not been accepted by the consumers. The CHANCE project also developed an innovative low-cost pizza with improved nutritional quality, materialized by increasing dietary fibers and protein contents and decreasing saturated fatty acid and cholesterol contents. The CHANCE pizza combined ingredients (i.e., soy paste, cooked ham fortified with vitamin D, whey cheese, and tomato sauce enriched with fibers isolated from food industry by-products) and solid technological features, in terms of rheological properties and dough elasticity, in order to meet nutritional and sensory properties tailored to a vulnerable group of consumers [115]. This example highlights that, enriching products with fibers and utilizing circular economy principles is essential in developing sustainable food products. These innovative foods are not UPFs per se. In another example, the NAMASTÈ project, novel ingredients based on the albedo citrus fibers were developed and used as stabilizers for fruit pulp suspensions and refrigerated food paste, making the products more homogeneous and attractive to consumers [109]. Furthermore, these were also used as ingredients to enrich transparent drinks with polyphenolic antioxidants, as well as in the formulation of textured food, and as vitamin-enriched fiber fillers for bakery products.

Beyond dietary fibers, several other compounds can be extracted from industrial by-products to fortify foods and optimize their nutritional value. For instance, PATHWAY-27 [116] is a project dedicated to developing novel foods that, when consumed at effective daily doses, can lower fasting concentrations of triglyceride and cholesterol in the blood. The added value was to use a natural powder extracted from red grape skins, a by-product of the wine industry, as a source of anthocyanins. The technological results were very positive, as the processing and the formulation allowed to recover at the end of the baking more than 90% of the bioactive compound added with the ingredients. Processing technologies thus made it possible to utilize by-products of the wine industry, otherwise destined for disposal and waste, while the extracts were included in foods with resulting satisfactory sensory characteristics appreciated by the consumers.

Successful circular economy strategies can also entail examples of product categories that are generally considered to be linked to chronic diseases, for example, extruded snacks. Food industry by-products such as fruit and vegetable pomace and bagasse, oilseed cakes, brewers spent grains, cereal brans and whey, can be used as excellent sources of nutritionally-enhanced and eco-friendly compounds in these products [117]. Leaves, seeds, peels or unused pulp can also become a valuable source of nutritional compounds, including essential oils with recognized antioxidant and antimicrobial properties, which can be used as natural additives in packaging applications [118].

The above examples demonstrate that sustainable integrated valorisation of food processing by-products to produce novel, healthy food products (that may or may not be classified today as UPFs) has already been tested with promising results; and can thus have a key role in facilitating access to foods meeting the nutritional requirements of individuals for health promotion and the global needs for sustainability. Although the circular economy frame for UPFs is still currently a niche concept, the overall wider role for circular economy principles and processing technologies to valorise by-products for sustainable food chains is necessary to meet the “Green Deal” and the healthy “farm-to-fork” strategies. As more and more bioactive molecules are discovered within raw materials, usually not reaching effective concentrations, the ability of processing technologies to separate, concentrate and incorporate these ingredients in foods becomes fundamental. These examples also demonstrate that utilizing circular economy principles and sustainable processes in food production do not necessarily result in products that are UPFs. It is therefore clear that sustainability and circular economy should be considered and included in the reformulation of the UPF concept and definition.

## 6. Conclusions

The putative adverse health effects of UPFs have been emphasized in recent years, associating negative connotations to whole groups of products and processing techniques, without properly considering the effects of food processing conditions, as well as several other internal (e.g., whole food composition, bioavailability, microbiome) and external (e.g., sociodemographic) factors. The complexity and the predicted immense size of this task calls for new paradigms and open innovation mentality and a new mindset promoting multidisciplinary collaborations and partnerships. The involvement of industry partners in medical research is ubiquitous [119] but, particularly when it comes to food and nutrition related research, this is often widely perceived by the public as a source of bias for the integrity of the research [120]. Therefore, a forward-looking perspective on the food industry involvement and science communication is urgently needed. The alliance between academia and industry should be looked at as one of the cornerstones of public health promotion. A moral responsibility is necessary in such collaborations, since suspicions about possible incorrect or biased research hamper scientific advancements, delay or impede application of new technologies, and thereby prevent translation of scientific discoveries with beneficial effects into new policies [121]. New paradigms of food evaluation and assessment should be developed on several novel pillars—enginomics, signalling, and precision nutrition—taking advantage of available digital technologies and artificial intelligence. Consequently, research needs to be carried out to generate the required scientific evidence to either expand the current or create new food evaluation and classification systems, incorporating all processing aspects that may have a significant impact on health and wellness, together with factors related to the personalization of foods and diets, as well as factors related to sustainability and circular economy. Facilitating access to foods that meet consumer expectations and demands in an affordable and convenient way, while simultaneously addressing planetary sustainability and individual nutritional needs, should be emphasized.

## Data Availability

Not applicable.
